# The Family Talk Intervention in Pediatric Oncology: Potential Effects Reported by Parents

**DOI:** 10.3390/children11010095

**Published:** 2024-01-12

**Authors:** Maria Ayoub, Camilla Udo, Kristofer Årestedt, Ulrika Kreicbergs, Malin Lövgren

**Affiliations:** 1 School of Health and Welfare, Dalarna University, 791 88 Falun, Sweden; cud@du.se; 2 Department of Health Care Sciences, Palliative Research Centre, Marie Cederschiöld University, 116 28 Stockholm, Sweden; u.kreicbergs@ucl.ac.uk (U.K.); malin.lovgren@mchs.se (M.L.); 3 Center for Clinical Research Dalarna, Uppsala University, 791 82 Falun, Sweden; 4 Department of Health and Caring Sciences, Faculty of Health and Life Science, Linnaeus University, 352 52 Växjö, Sweden; kristofer.arestedt@lnu.se; 5 Department of Women’s and Children’s Health, Childhood Cancer Research Unit, Karolinska Institute, 171 77 Solna, Sweden; 6 Louis Dundas Center, Great Ormond Street Institute of Child Health, University College London, London WC1N 1EH, UK; 7 Advanced Pediatric Home Care, Astrid Lindgren Children’s Hospital, Karolinska University Hospital, 171 64 Solna, Sweden

**Keywords:** Family Talk Intervention, pediatric oncology, psychosocial support, family, parents

## Abstract

Background: Childhood cancer impacts the family system and has psychosocial consequences for all family members. For the parents, the ill child, and the siblings to be able to adjust to this challenging situation, the whole family needs access to psychosocial support. However, only a few such family interventions in pediatric oncology have been evaluated. The aim of this study was to explore the potential effects of a family-centered intervention, the Family Talk Intervention (FTI), in pediatric oncology from the parents’ perspectives. Methods: A concurrent mixed methods design was used for this study. Data were derived from a pilot study of 26 families recruited from one pediatric oncology center in Sweden. This study focused on questionnaire and interview data from 52 parents. Results: After participation in FTI, the parents felt more satisfied with the conversations within the family about the illness. FTI also contributed to strengthened family togetherness, including more open communication and improved family relations, as described by the parents. Parents further expressed that they felt more empowered in their parenting role following FTI. Conclusions: The findings regarding FTI’s ability to improve family communication and family relations, thus strengthening family togetherness in families with childhood cancer, are promising. This provides motivation for a large-scale study of FTIs in pediatric oncology.

## 1. Introduction

Families affected by childhood cancer experience various forms of illness-related stressors that have psychosocial consequences for all family members [1]. Parents must cope with different challenges, such as seeing their children suffer from the side effects of the illness, a high frequency of hospitalizations and treatments, the fear of possible relapse, and the caregiving demands [2,3]. Many parents struggle with worries about not providing adequate care for their children and neglecting the needs of siblings [2]. They also find it hard to communicate with their children about the illness [4]. These situations contribute to a high level of stress and many parents experience a deterioration of their own physical and psychosocial well-being [5,6]. Parental distress has a negative impact not only on the parents, but also on family life satisfaction and family relationships [7].

A family’s ability to communicate their concerns, emotions, and thoughts is a protective factor and functions as a key component of family resilience [8]. In families affected by childhood cancer, the activation of resilience processes helps overcome family distress and achieve balance in family functioning [9,10]. Supporting family functioning, including open communication, is therefore crucial for maintaining psychosocial well-being in these families [11]. There are numerous types of psychosocial interventions for families of children with cancer, such as problem-solving skills training (PSST), cognitive behavioral therapy (CBT), and family therapy [12]. Despite the recommendations for psychosocial family interventions, which target the family unit as well as each single family member, and the pivotal role they can play, only a few such interventions have been systematically evaluated in pediatric oncology [13,14].

The Family Talk Intervention (FTI) is a family-centered intervention that includes the whole family. It was originally developed in psychiatric care to support families in which a parent is suffering from depression [15]. The aims of FTI are to facilitate family communication about the illness and related subjects, to support parenting in making the children’s needs visible, and to help the families realize their strengths and how best to use them [16]. FTI has an eclectic approach, including psycho-educational, narrative, and dialogical theories, and its primary focus is on the children [17]. FTI has been shown to be helpful for families in psychiatric and somatic care when a parent with dependent children is ill; it has been found to increase family communication, to strengthen family relations [18], and to improve the family’s psychosocial health [19,20]. Since the main goals of FTI could respond to the psychosocial support needs reported by families in pediatric oncology, FTI has been pilot-tested and evaluated in this context [21]. It has been proven feasible by families in pediatric oncology in terms of acceptability. FTI has also been shown to be effective in various ways from the children’s perspectives [22]. Given the salient role of the parents, parenting satisfaction is thus an important factor for family adaptation and well-being when a child has cancer. However, knowledge about how the parents in the participating families view FTI is limited. This study therefore aims to explore the potential effects of FTI in pediatric oncology from the parents’ perspectives.

## 2. Materials and Methods

### 2.1. Design

This study originates from a pilot study of a complex intervention [23] in pediatric oncology using data from parental questionnaires and interviews [21]. To explore the potential effects of FTI as reported by the parents, a concurrent mixed methods design [24] was used in which quantitative and qualitative data were triangulated. The use of two different methods was a pragmatic choice to increase confidence in results reached. The two data sets were analyzed independently and the results were interpreted together by identifying common concepts across both sets of findings.

### 2.2. The Family Talk Intervention

FTI is manual-based and involves six meetings with family members in various configurations. An overview of FTI meetings is presented in Table 1. The meetings are performed at 1–2-week intervals and led by FTI-educated clinicians, in this study described as interventionists (two social workers and one deacon), working in pairs. The meetings are held in a location decided on by the family.

### 2.3. Study Population

Eligible families were those with an ill child who was enrolled at one pediatric oncology center in Sweden, 2–3 months after diagnosis or relapse, during a period of one year (September 2018 to August 2019). Participating families had to understand and speak Swedish. Either the entire family or part of the family could participate, but the minimum was that one parent and one child (ill or healthy), aged 6–19 years, had to be included. Contact nurses at the clinic identified families that met the inclusion criteria (n = 61). Of the families identified, 27 consented to participation and 26 completed the intervention (52 parents, 26 ill children, and 37 siblings). The families were given verbal and written information about the study. Written informed consent for participation was obtained from the parents/guardians of children under 15 years. Parents and children aged 15 years and over gave their own written informed consent, in accordance with Swedish law.

The study was approved by the Regional Ethical Review Board in Stockholm (No. 2018/250-31/2, 5 March 2018 and 2018/1852-32, 1 October 2018).

### 2.4. Data Collection

Data were collected using questionnaires completed by all family members at baseline (before FTI started), in conjunction with the end of FTI (follow-up 1), and six months after the completion of FTI (follow-up 2). In addition, interviews with all participating family members were conducted in conjunction with the end of FTI. This study focused on data from the parents’ responses to the questionnaires completed at all three timepoints and interviews with the parents.

#### 2.4.1. The Questionnaires

Web-based questionnaires were used to collect data. Questions regarding demographic characteristics, psychometrically validated instruments, and study-specific questions were used. These covered the primary outcome of family communication, and the secondary outcome of psychosocial health including resilience, quality of life, family satisfaction, grief experiences, and knowledge about the illness. In this study, questions regarding family communication were analyzed to examine the potential effects of FTI from the parents’ perspective. Questions concerning family satisfaction and resilience were also analyzed to examine the impact of FTI on the families’ psychosocial health.

##### Family Communication

The 10-item Family Adaptability and Cohesion Scale IV (FACES IV), Family Communication, was used [25]. This instrument focuses on the exchange of information, both factual and emotional. It captures constraints and the degree of understanding and satisfaction experienced by family members in family communication interactions [26,27]. Family members rate their family communication using a five-point response scale. The response options range from 1 “strongly disagree” to 5 “strongly agree”. The scale score is calculated by summing the responses, giving a possible range of 10 to 50. According to the manual [28], the scores are categorized into very high (44–50), high (38–43), moderate (33–37), low (29–32), and very low (10–28) family communication. The internal consistency of the scale has been reported to be high with a Cronbach’s alpha of 0.90 [28].

In addition, study-specific questions about illness-related communication were included. For example: “I would like to talk more about my child’s illness to someone in my family” (see all items in Table 2). These questions were rated using a rating scale ranging from 1 “strongly disagree” to 4 “strongly agree”. The study-specific questions have been used in earlier FTI studies [18] and tested on five families in pediatric oncology, which resulted in some minor changes.

##### Family Satisfaction

Family satisfaction was measured using the FACES IV, Family Satisfaction. This is a 10-item scale that assesses the degree of satisfaction family members have within the family, including closeness and flexibility [25]. Family members rate their level of satisfaction on a five-point response scale ranging from 1 “strongly disagree” to 5 “strongly agree”. The scale score for family satisfaction is calculated by summarizing the responses, generating a possible range of 10 to 50. The scores are categorized into very high (44–50), high (38–43), moderate (33–37), low (29–32), and very low (10–28) family satisfaction. For this scale, the internal consistency has been reported with a Cronbach’s alpha of 0.92 [28].

In conjunction with the questions about family satisfaction, study-specific questions about parenting were also asked. Parents rated their satisfaction with their parenting on a five-point rating scale ranging from 1 “very dissatisfied” to 5 “extremely satisfied”.

##### Resilience

Resilience among the parents was measured using the short version of the Resilience Scale (RS-14) [29,30]. The RS-14 comprises 14 items and covers five core elements of resilience: self-reliance, purpose, equanimity, perseverance, and authenticity. Each item is rated on a seven-point scale. The response options range from 1 “strongly disagree” to 7 “strongly agree”. The scale score is calculated by summarizing the responses, giving a possible range of 14 to 98. According to the manual, the scores are categorized into very low (14–56), low (57–64), moderately low (65–73), moderate (74–81), moderately high (82–90), and high (91–98) resilience. The internal consistency of this scale has been reported with a Cronbach’s alpha of 0.93 [31].

#### 2.4.2. Interviews

The interviews were guided by a semi-structured interview guide. They were conducted by members of the research group and held in the families’ homes with two exceptions (one at the hospital and one digitally due to the COVID-19 pandemic). Each interview focused on the parents’ experiences of participating in FTI through questions such as “Could you tell me about your experiences of participating in the support program?”, “What was good/less good?”, and “Could you tell me about your conversations with each other during/after the support program?”

The interviews were audio-recorded and lasted between 18 and 84 min (median: 45 min). The parental couples were interviewed together with one exception (one parental couple asked for separate interviews).

### 2.5. Data Analyses

#### 2.5.1. Statistical Analyses

Descriptive statistics were used to present sample characteristics and study variables. Continuous data were presented as mean and standard deviations, ordinal data as median and quartiles, and non-ordered categorical data as frequencies and proportions. To handle the ordinal nature of the data for the outcome variables, Friedman’s test was used to examine the changes over time reported by parents regarding the family communication scale, family satisfaction scale, and resilience scale, and the study-specific questions from baseline and follow-ups. The post hoc test was Bonferroni-corrected. Kendall’s W was used to estimate the overall effect size and interpreted as: small effect (0.1 - < 3), moderate effect (0.3 - < 0.5), and large effect (≥0.5). Overall, the level of statistical significance was set at *p* < 0.05. All analyses were conducted in R 4.3.0 (R Foundation for Statistical Computing, Vienna, Austria) and Rstudio 2023.03.1+446 (PBC, Boston, MA, USA) including the following packages: dplyr 2.3.2, psych 2.3.3, rstatix 0.7.2, and summarytools 1.0.1.

#### 2.5.2. Thematic Network Analysis

The interviews were transcribed verbatim and analyzed with thematic network analysis, a technique for conducting thematic analyses by creating basic, organizing, and global themes of the qualitative material [32]. Each interview was read several times and the material was coded by the first author (M.A.). Codes were documented on a coding sheet and critically discussed by all authors. The coded text segments were grouped into basic themes and clustered into organizing themes. Thereafter, the organizing themes were grouped into one global theme, representing the core of the analysis. All authors were involved in the analysis and the process was considered complete when the authors reached an agreement regarding the potential effects of FTI.

## 3. Results

### 3.1. Sociodemographic Characteristics of Parents

A total of 52 parents, from 26 families, participated in this study: 29 mothers and 23 fathers (Table 3). Their mean age was 44 years (34–64) and most of the parents had an academic degree (n = 35, 67%). The majority lived in a nuclear family configuration (n = 21, 81%), with one to three children. Half of the ill children were diagnosed with a central nervous system tumor (n = 13, 50%) and three children had relapsed when enrolled into the study (not reported in the table). At follow-up 2, six months after FTI had end, 4 of the 26 children had died.

### 3.2. Potential Effects of FTI

#### 3.2.1. Quantitative Results

Table 3 presents the score distribution and differences over time for the family communication scale, family satisfaction scale, resilience scale, and the study-specific questions regarding family communication and satisfaction. Parents reported low to moderate family communication and family satisfaction, and moderate levels of resilience at baseline. No significant changes were shown for any of these outcomes after participation in FTI (Table 2).

A significant difference over time was found for the study-specific question “I would like to talk more about my child’s illness with someone in my family” (*p* = 0.041). The post hoc test showed that the parents scored significantly lower in their needs to communicate more about the child’s illness within the family at the second follow-up, compared to the baseline assessment (*p* = 0.036), but the effect size was below minor, rather than small (*W* = 0.09).

There was also a significant difference over time for the study-specific question “I am satisfied with the conversations in the family about my child’s illness” (*p* = 0.014). The post hoc test showed that the parents scored significantly higher satisfaction with the conversations within the family about the child’s illness at the first follow-up compared to the baseline assessment (*p* = 0.01), and at the second follow-up compared to the baseline assessment (*p* = 0.034), but the effect size was small (*W* = 0.12).

#### 3.2.2. Qualitative Results

**Figure 1:**
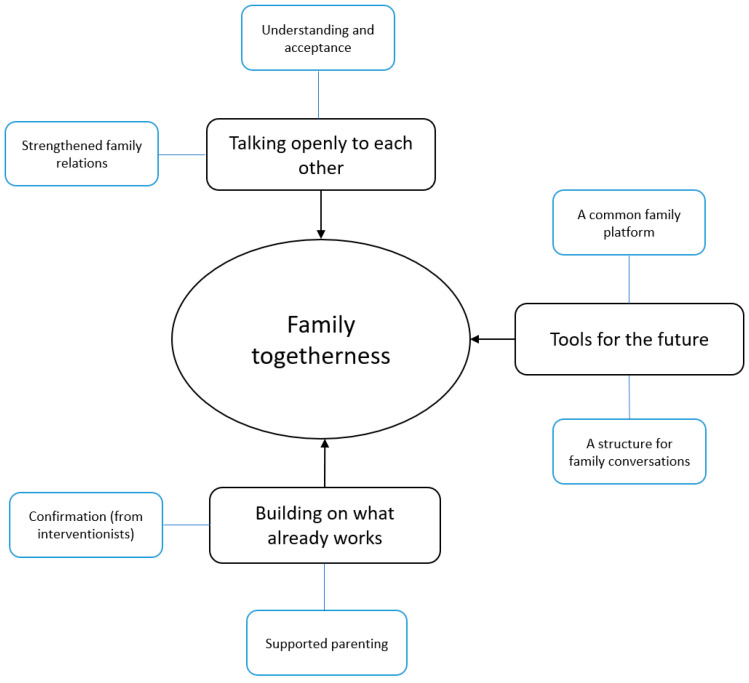
Networks of themes: family togetherness.

The analysis of the interviews resulted in a network consisting of six basic themes, three organizing themes, and one global theme that captured the central points of the interviews (Figure 1).

##### Family Togetherness

Overall, the parents experienced that family togetherness had been strengthened after participation in FTI. Parents’ descriptions showed that FTI appears to have contributed to open family communication and therefore improved family relations. Participation in FTI also provided the families with ideas and strategies, which the parents described as tools they could use as a family to better manage everyday life and challenging situations. These tools included, e.g., a common family platform and structure for family conversations. Continuing to build on the existing strengths within the family that were identified in FTI also promoted family togetherness (Table 4).

##### Talking Openly to Each Other

Parents stated that participation in the intervention had contributed to increased openness within the family, i.e., FTI supported more open communication. In addition, they described increased understanding and acceptance, and closer relationships, between family members. Most of the parents stated that they were able to talk more openly with each other about illness-related subjects and how they felt in relation to the child’s illness. Parents also described that they now felt encouraged to have difficult conversations with their children, something they had previously often avoided.

Following the intervention, parents expressed a greater acceptance and understanding of each other’s different perspectives, wishes, and needs. Talking more openly about feelings and thoughts within the family had, according to the parents, contributed to strengthened relationships between individual family members. Further, some of the parents stated that the improved family relations affected the family dynamics positively and that they felt more satisfied with family life in general. Some parents reported that they now spend more time as a family and do everyday activities together more often compared to before FTI.

While most parents described an increased openness in family communication, some expressed that they had also become more aware of challenges within the family and the issues that different family members were struggling with.

##### Tools for the Future

The parents described feeling more prepared to deal with potential upcoming challenging life events after participating in FTI. Some of the parents said that the interventionists provided them with suggestions and concrete tools, such as communication cards, to support them as parents in communicating with their children. Others expressed more generally how regular family meetings, such as FTIs, provided a framework for the family to adhere to and continue to use. The parents described how FTI had inspired them to create a forum where, as a family, they could come together to discuss any disagreements, schedules, routines, or other topics a family member might be concerned about. These family meetings provided an opportunity to coordinate and communicate important issues. The parents further expressed that such weekly family gatherings have contributed to fewer family conflicts and helped foster a sense of responsibility and mutual cooperation.

While most parents stated that FTI had influenced the family to continue the dialogue with each other, they also expressed concern regarding how to maintain the open conversations. However, some parents expressed a feeling of hope that by using the tools learned from FTI, the family would gradually return to a “normal” everyday life and family health.

##### Building on What Already Works

Being a parent of a child with cancer was deeply challenging and it also brought with it great responsibility for keeping the family together. Some parents expressed often feeling inadequate as a parent. They stated, however, that participation in FTI had to some extent helped them overcome feelings of inadequacy in their parenting, for example by being acknowledged and encouraged as parents. The parents expressed that they now felt more confident in how to manage and support the ill child’s and their sibling’s individual needs. Further, receiving positive feedback from interventionists regarding their parental role was described as strengthening and valuable.

The parents also expressed the value of being acknowledged and encouraged to continue what they were already doing well and build on what already worked. One parent described that, after participation in FTI, they felt motivated as parents to continue engaging the whole family in order to maintain these already well-functioning processes and keep moving forward.

## 4. Discussion

In this study, the potential effects of using FTI in a pediatric context are reported. After participating in FTI, the parents reported feeling more satisfied with the conversations held within the family and expressed less need for more communication with someone in the family about their child’s illness. They also reported that FTI contributed to increased family togetherness, including open communication and family relations.

The findings of this study are similar to those reported from earlier FTI studies in other care contexts, for example when a parent has an affective disorder [19,20,33,34] or a severe illness such as cancer [18], in terms of a better understanding of the illness, reduced worry, and decreased family conflicts. Most of the parents in the present study reported that FTI contributed to improved open family communication about the situation and thereby strengthened family relations, which is in line with reports from families in adult care [35,36].

Supporting families in coping with the psychosocial aspects of a child’s cancer diagnosis has shown positive effects on family functioning, including enhanced family ties [37]. When considering the support obtained from FTI, almost all parents stated in their interviews that family togetherness had in some way been strengthened and that they now felt more satisfied with family life in general. Similar to these findings, siblings in families affected by childhood cancer reported that family relationships had been strengthened following FTI [22]. Taken together, these results imply that FTI might have initiated a process within the family that has contributed positively to overall family functioning.

Increased satisfaction with family communication following FTI was shown in both the quantitative and qualitative data. Promoting family communication is one of the central goals of FTI [16] and effective family communication is a key aspect for family functioning [38]. The present findings indicate the ability of FTI to facilitate family members openly sharing experiences with each other, thus contributing to strengthened family relations and family functioning. Although a cancer diagnosis may affect all family members negatively, it may also bring families closer together [39,40]. The findings should, however, be considered with caution, since the child’s cancer diagnosis itself may have brought the family closer together, not just participation in FTI.

Furthermore, open communication processes within families are a key component for building family resilience [8], which is another main goal of FTI [16]. Although no significant differences were found for resilience in the quantitative data set following FTI, interview data indicated otherwise. Specifically, factors related to resilience had somewhat improved in the families after their participation in FTI. This is in line with previous studies demonstrating that professionals play an important role in helping these families to recognize their own strengths [5,9]. Considering that the findings are based on parental data, it could be argued that these findings reflect parental resilience more than family resilience. However, the findings from the interview data seem to involve management on a family level rather than how the parents themselves cope with their own emotional reactions.

Parents expressed that parenting a child with cancer and at the same time keeping the family together was challenging and sometimes exhausting. However, participation in FTI seemed to empower the parents in their role as parents and helped reduce feelings of inadequacy. Parental satisfaction has been found to be a potential resource for improving the psychosocial well-being of parents of children with cancer [7] and thus contribute positively to overall family functioning [10,37]. According to our results, participation in FTI seems to be a way of strengthening the parents, which is crucial not only for them as individuals, but also for family functioning.

A strength of this study is its mixed methods design, which included both quantitative and qualitative data. This provided a fuller picture of the findings, since the rich data from parental interviews were used to complement quantitative outcomes, and to build upon the non-significant findings. Although the quantitative results, where psychometric instruments were used, were most often not significant, some of the study-specific questions showed significant findings. This might be related to the fact that the study-specific questions were more sensitive to what FTI can affect, as the study-specific questions were developed based on interviews with families that have participated in FTI in other care contexts [18]. Even if this study presents promising results, it has several limitations. The small sample size implies reduced statistical power and therefore an increased risk of type II errors. Another limitation is that the sample consisted of mostly highly educated Swedish nuclear families. This is a common challenge when conducting intervention research; namely, finding representative participants and targeting those from lower socioeconomic backgrounds [41]. Consequently, the findings should be generalized with care to families with less well educated parents.

In summary, the findings of this study are promising regarding FTI’s ability to facilitate family communication and relations, and thus strengthen family togetherness, which most likely can support resilience in families with childhood cancer. Since this study indicates that FTI constitutes a much-needed psychosocial support intervention, further large-scale studies are encouraged before recommending that FTI be implemented broadly. In the future, it would be appropriate to include clinicians from the practice, rather than having FTI performed by external interventionists, as in the pilot study, as this could facilitate continuity. This study might also provide a good basis to help refine the design and methodology of future studies.

## Figures and Tables

**Table 1:** Family members involved and the focus of each meeting in FTI.

Meeting 1–2: with the Parents	Meeting 3: with each Child	Meeting 4: with the Parents	Meeting 5: with the whole Family	Meeting 6: with the Parents or the whole Family	Meetings 7–11
The parents’ stories. Setting goals for the intervention	Each child’s story. The child’s understanding of the illness and the situation. The child’s worries and questions.	Summary of worries and questions from Meeting 3. Planning ‘the family talk’ (Meeting 5).	‘The family talk’. Preferably led by the parents and covering issues raised by both children and parents.	Follow-up with a focus on how to communicate within the family in the future to achieve the family’s goals.	Extra meetings if needed.

**Table 2:** Score distribution, differences over time, and effect size regarding communication within the family, satisfaction with family life, and resilience from baseline to follow-up 1 (in conjunction with the end of the intervention) and follow-up 2 (6 months later) (n = 52).

	Score Distribution, Mdn (q1–q3)					
	Baseline	Follow-Up 1	Follow-Up 2	*p*-Value ^a^	ES ^b^	Post-hoc Test ^c^
Family communication, n = 41	36 (32–40)	36 (32–40)	35 (30–40)	0.093	0.06	- - C
Family satisfaction, n = 41	30 (25–37)	30 (26–34)	29 (24–36)	0.545	0.01	- - -
Resilience, n = 41	77 (66–86)	79 (68–83)	77 (66–84)	0.334	0.03	- - -
I would like to talk more about my child’s illness to someone in my family, n = 34	2 (1–2)	1 (1–2)	1 (1–2)	0.041	0.09	- B -
I am satisfied with the conversations in the family about my child’s illness, n = 35	3 (2–3)	3 (3–4)	3 (3–4)	0.014	0.12	A B -
I want to show more how I feel to someone in my family, n = 39	2 (1–2)	1 (1–2)	1 (1–2)	0.538	0.02	- - -
How satisfied are you with: your parenting of your child with cancer, n = 35	3 (3–4)	4 (3–4)	4 (3–4)	0.499	0.02	- - -
How satisfied are you with: your parenting of your other children (if there are siblings), n = 35	3 (2–4)	3 (2–4)	3 (3–4)	0.492	0.02	- - -

^a^ Friedman’s test; ^b^ Kendall W effect size: small effect 0.1 - < 0.3, moderate effect 0.3 - < 0.5, large effect ≥ 0.5; ^c^ Bonferroni-corrected post hoc test: significant differences are presented with an A (baseline vs. follow-up 1), B (baseline vs. follow-up 2), and/or C (follow-up 1 vs. follow-up 2); Note. n = 36–47 due to internal missing data.

**Table 3:** Sociodemographic characteristics for the families (n = 26) participating in FTI.

		Parents n = 52	Ill Children n = 26	Siblings n = 37
**Gender, n (%)**				
	Female	29 (56)	14 (54)	20 (54)
	Male	23 (44)	12 (46)	17 (46)
**Age (years), M (SD), n (%)**		44 (6.6) (34–64)	10 (3.9) (1–17)	11 (5.3) (3–24)
**Educational level, n (%)**				
	University	35 (67)		
	High school	15 (29)		
	Elementary school	0		
	Other	2 (4)		
**Cancer diagnosis (according to parents**)				
	Central nervous system tumor		13 (50)	
	Lymphoma		5 (19)	
	Leukemia		4 (15)	
	Sarcoma		2 (8)	
	Other		2 (8)	

**Table 4:** Parents’ experiences of the Family Talk Intervention (interview data).

Family Togetherness	Statements from the Interviews
**Talking openly to each other**	
Understanding and acceptance	But since then it has become much better because you and I have gained a better understanding of each other, because we think very differently and do things in very different ways. And that has benefited the family enormously as well. *(Mother in a nuclear family with one ill child and two siblings)*
Strengthened family relations	you [stepmum and sick child] have had very good conversations...And where you [stepmum] have really listened and taken things to heart and you’ve found compromises and so on, and I think you’ve strengthened your relationship through that. *(Father in a stepfamily with one ill child and one sibling)*
**Tools for the future**	
A common family platform	we decided that we would have a dinner meeting on Sundays. And it was the children’s suggestion that it should be in connection with dinner. So that, you know, then everyone is together, and anyone can bring things up that they... well, good things, but also things that we need to think about or improve on. *(Mother in a nuclear family with one ill child and one sibling)*
A structure for family conversations	it’s been, you know, a clear, a good forum for us even though we’ve talked a lot all the time it’s felt like in these conversations we’ve been able to start from when we’ve continued to talk, as a family too *(Mother in a nuclear family with one ill child and two siblings)*
**Building on what already works**	
Confirmation (from interventionists)	We’ve gained two insights, one of which is that we have an unusually good conversational climate in our family. It was great to hear. Because it doesn’t always feel that way […] So we felt a lot stronger, we felt encouraged to continue on the same path *(Mother in a nuclear family with one ill child and one sibling)*
Supported parenting	that we got to hear, like: “Yes, but we are doing something good” because it’s so easy to think that... I don’t know, it’s always this feeling of inadequacy. But it’s nice to hear from others that: “Yes, but you are being really strong and you are doing great things”, and it’s also important to feel, to feel stronger in yourself *(Mother in a nuclear family (nr. 19) with one ill child and one sibling)*

## Data Availability

The data presented in this study are available on request from the corresponding author. The data are not publicly available due to restrictions of privacy and ethical.
